# The safety and efficacy of esketamine in comparison to dexmedetomidine during drug-induced sleep endoscopy in children with obstructive sleep apnea hypopnea syndrome: A randomized, controlled and prospective clinical trial

**DOI:** 10.3389/fphar.2022.1036509

**Published:** 2022-12-01

**Authors:** Zheng Yongping, Li Xinyi, Sang Aming, Xie Qiang, Zhou Tianqi, Shen Mengmeng, Chen Xiong, Song Xuemin

**Affiliations:** ^1^ Department of Anesthesiology, Zhongnan Hospital of Wuhan University, Wuhan, Hubei, China; ^2^ Department of Otorhinolaryngology-Head and Neck Surgery, Zhongnan Hospital of Wuhan University, Wuhan, Hubei, China; ^3^ Postanesthesia Care Unit, Department of Anesthesiology, Zhongnan Hospital of Wuhan University, Wuhan, Hubei, China; ^4^ Department of Anesthesiology, Research Centre of Anesthesiology and Critical Care Medicine, Zhongnan Hospital of Wuhan University, Wuhan, Hubei, China

**Keywords:** dexmedetomidine, esketamine, drug-induced sleep endoscopy (DISE), obstructive sleep apnea hypoapnea syndrome, pediatrics

## Abstract

**Background and Purpose:** Data and high-quality studies of anesthetic methods for children with obstructive sleep apnea hypopnea syndrome (OSAHS) who undergo drug-induced sleep endoscopy (DISE) are limited. Research on pediatric DISE using esketamine has never been reported before. To test the safety and efficacy of esketamine during DISE in children with OSAHS, we compare esketamine (Group K) with dexmedetomidine (Group D) in this study.

**Methods:** 100 children with ASA Ⅰ∼Ⅱ grade, prepared for an elective adenotonsillectomy under general anesthesia, were enrolled in this study and randomized into two groups. Midazolam 0.1 mg/kg was administered intravenously for both groups. In Group D a 1 μg/kg bolus of dexmedetomidine was given over 10 min followed by the infusion rate 1 μg/kg/hr to the end of DISE. Group K received a 1.0 mg/kg IV bolus of esketamine over 10 s followed by the infusion rate 1 mg/kg/hr to the end of DISE.

**Results:** Group K had a higher percentage of success than Group D (*p* = 0.008). The onset time of Group K was shorter than that of Group D (*p* = 0.000). The University of Michigan Sedation Scale (UMSS) score of Group K was higher than that of Group D (*p* = 0.005). The risk of adverse effects (AEs) was lower in Group K (*p* = 0.000). In Group D, systolic and diastolic blood pressure (SBP and DBP) and heart rate (HR) all decreased, while in Group K, SBP, DBP, and HR hardly changed.

**Conclusion:** Esketamine in comparison to dexmedetomidine provides more effective and safer depth of anesthesia for OSAHS pediatric DISE by ensuring short onset time, deep sedation, and few AEs.

**Clinical Trial Registration**: ClincalTrials.gov, identifier NCT04877639

## Introduction

Obstructive sleep apnea hypopnea syndrome (OSAHS) is generally believed to be a common health problem (Li et al., 2010). 2%–4% of children with OSAHS are associated with a substantial morbidity (Park et al., 2011), including failure of growth, impaired neurocognitive and neurobehavioral abnormalities (Miano et al., 2011), systemic hypertension, pulmonary hypertension (Goldbart et al., 2010), cor pulmonale, etc (Kelly et al., 2010). A great number of children with OSAHS are cured after adenotonsillectomy. However, 10%–20% of children have continuing symptoms after surgery (Bhattacharjee et al., 2010). One of the reasons for uncured OSAHS is failure to identify all sites of the upper airway obstruction (Marcus et al., 2012).

There has been increased interest in using drug-induced sleep endoscopy (DISE), an emerging endoscopic technology, to assess upper airway obstruction in patients with OSAHS in a sleep-like state induced and maintained by anesthetic drugs. Using a flexible nasal endoscope, DISE locates the sites and patterns of airway collapse accurately, predicts the benefit of surgery, and customizes a targeted surgical approach for each patient (Capasso et al., 2016).

To mimic physiological sleep with decreased oxygen saturation levels, the ideal anesthetic administration during DISE should involve the use of titrable pharmacological agents with short biological half-life and minor influence on muscle tone and respiratory drive (Liu et al., 2020). Dynamic evaluation of DISE in children with OSAHS is often achieved by using sedatives and anesthetics including benzodiazepines, pentobarbital, remifentanil, propofol, ketamine, dexmedetomidine, and their combination (Cho et al., 2015; Liu et al., 2020). Propofol acts through the inhibitory neurotransmitter GABA to diminish behavioral responsiveness as if in a state of non-rapid eye movement (NREM) sleep (Murphy et al., 2011). It may compromise the airway due to muscular flaccidity and respiratory drive suppression. As a selective alpha-2 adrenergic agonist, dexmedetomidine is a highly recommended agent for DISE owing to its analgesic, amnesic and anxiolytic characteristics. Ehsan reviewed and concluded that drugs such as dexmedetomidine have the least impact on respiratory control and may be most effective in DISE (Ehsan et al., 2016). Though dexmedetomidine successfully induces sedation for non-invasive procedures, it does not provide sufficient depth of anesthesia when used as a sedative/anesthetic alone for invasive procedures (Mahmoud and Mason, 2015). Based on his review, Liu thought that the optimal scheme might be made by combining ketamine with dexmedetomidine (Liu et al., 2020). As a non-competitive n-methyl-d- aspartic acid (NMDA) receptor antagonist, ketamine offers good analgesia and amnesia with natural respiratory pattern, however, its role as a sedative has been restricted by the occurrence of vomiting and psychomimetic side effects (Sruthi et al., 2018; Wang et al., 2019). The substance ketamine is a racemate comprising two enantiomers─mirror-like configurated molecules S (+)- and R (−)- ketamine. Compared to both the racemic and R (−)-ketamine, S (+)-enantiomer demonstrated the greater efficacy with lower dosage in experimental studies on animals (Schmidt et al., 2005).

Children with OSAHS are vulnerable to upper airway obstruction during sedation and anesthesia because they are more sensitive to the respiratory inhibitive effects of hypnotics and sedatives. We need to avoid using airway intervention to improve airway patency for them. Thus, it poses a challenge to obtain perfect dynamic airway assessment during DISE for these patients. It is urgent and critical to find good anesthetic drugs for their DISE. Data and high-quality studies of anesthetic methods for OSAHS pediatric DISE are limited (Liu et al., 2020). Research on pediatric DISE using esketamine has never been reported before. To test the safety and efficacy of esketamine during DISE in children with OSAHS, we compare esketamine (Group K) with dexmedetomidine (Group D) in this study.

## Materials and methods

### Ethics approval

This study, approved by the Ethical Board for Clinical/Scientific Research Project of Zhongnan Hospital of Wuhan University (Approval Number: 2021071), was conducted in accordance with the International Conference on Harmonization Guidelines for Good Clinical Practice and the Declaration of Helsinki. Trial Registration: ClincalTrials.gov. Identifier: NCT04877639. Chinese Clinical Trial Registry: http://www.chictr.org. Number: ChiCTR2100045914.

### Study design, setting and population

100 children with ASA Ⅰ∼Ⅱ grade who are prepared for an elective adenotonsillectomy under general anesthesia were enrolled between 17 May 2021 and 22 November 2021 at Department of Otorhinolaryngology⁃Head and Neck Surgery of Zhongnan Hospital of Wuhan University in the study. Inclusion criteria included 1) 3–12 years old, and 2) informed consent from subjects’ legal guardian. Exclusion criteria were 1) ASA physical status>Ⅲ, 2)a baseline oxygen saturation<95%, 3)Body Mass index (BMI) > 30 kg/m^2^, 4)Mallampati score iv, 5) chronic heart/lung/liver/kidney diseases, iind-iiird degree a-v block, psychiatric illness, (6 drug abuse or history of chronic analgesic use, and 7) allergy against the study medications (dexmedetomidine or esketamine). The subjects were numbered according to their treatment order and randomized into two groups (Group D and Group K). Randomization was achieved by computer generated random numbers hidden in a sealed opaque bag. A nurse, who was not involved in the study, read the numbers and assigned two groups.

In the inpatient ward, an intravenous (IV) catheter was inserted for all children in the two groups. Midazolam 0.1 mg/kg was administered intravenously for both groups. In Group D a 1 μg/kg bolus of dexmedetomidine (U2102003, Hu Nan Ke Lun Pharmaceutical Co. Ltd., China) was given over 10 min followed by the infusion rate 1 μg/kg/hr to the end of DISE. Group K received a 1.0 mg/kg IV bolus of esketamine (210126BL, Jiang Su Heng Rui Pharmaceuticals, China) over 10 s followed by the infusion rate 1 mg/kg/hr to the end of DISE. The dexmedetomidine and esketamine were each diluted in a 50 ml syringe separately and labeled as infusion A and B respectively, and administered using syringe pumps (WZS-50F6 Double channel micro-infusion pump, Smiths medical, China) by an anesthesiologist, who covered all syringes and infusion sets as well as the screen of the syringe pumps by aluminum foil paper to assure blindness of the study.

With their heads remaining neutral, the patients in both groups were supine and could breathe spontaneously. Throughout the procedure the patients received continuous oxygen, and their pulse oximetry, electrocardiogram and blood pressure were monitored. The level of sedation was monitored with an A-2000 BIS monitor (BIS LoC 2 channel, BIS Complete Monitoring System, 2011 Covidien 11c, Singapore). After UMSS>3 and BIS 65–75, the nostrils, the nasopharynx, the oral cavity and the hypopharynx were checked by a flexible fibrous laryngoscope to ascertain airway obstructions. The base of tongue and supraglottic structures were also checked. The patients in both groups were given propofol 0.5 mg/kg when they moved during DISE. After DISE, intubation was employed and then mechanical ventilation was achieved by IPPV. An elective adenotonsillectomy under general anesthesia was performed at last.

### Observational index

Demographic and PSG data: Demographic and polysomnography (PSG) data, including age, gender, height, weight, American Society of Anesthesiologists (ASA) physical status, Body Mass Index (BMI), respiratory disturbance index (RDI), apnea hypopnea index (AHI), severity of OSAHS and Mallampati Score (Smith et al., 2020), were gathered.

Vital signs: Heart rate (HR), systolic blood pressure (SBP), diastolic blood pressure (DBP), electrocardiogram (ECG), respiratory rate (RR) and pulse oxygen saturation (%, SpO_2_) were collected before medication and DISE (T0), 5 min after medication and before DISE (T1), 1 min after start of DISE (T2), 1 min after completion of DISE (T3), 1 min after tracheal intubation (T4), 1 min after extubation (T5) and 30 min after extubation (T6).

Time for each procedure: Onset time, DISE time, operation duration, recovery time and residence time were recorded.

Percentage of success: The ratio of completed DISE cases and total cases was calculated.

UMSS score, BIS and ABJ score: Depth of sedation was evaluated by the University of Michigan Sedation Scale (UMSS) (Malviya et al., 2002; Haberland et al., 2011) at T1 and the bispectral index (BIS) (Ibrahim et al., 2001) at T0, T1, T2, T3, T4, T5, and T6. Awakening and behavior judgment score for newborns and children (ABJ score) (Pees et al., 2003) was recorded at T6.

Adverse effects (AEs) and corresponding treatments: AEs such as hypoxemia (SpO_2_<90%, Apnea>20s), laryngospasm, patient movement, abortion of examination, PONV, delirium and propofol rescue were observed during and after DISE. Hypoxemia was relieved by oxygen therapy. Laryngospasm was treated by the positive pressure ventilation and/or administration of propofol. Propofol 1.0 mg/kg was used for delirium or uncontrolled movements. 0.15 mg/kg of Ondansetron and/or 0.25 mg/kg of dexamethasone (below 10 mg) were given for postoperative nausea and vomiting (PONV) (Martin et al., 2019). Other AEs were minor and transient, which needed no special treatment.

### Statistical analysis

Power analysis performed by using the nQuery Advisor with an inter-group difference of 20.4 and a standard deviation of 23, α = 0.05, *ß* = 0.2 (power = 80%), assuming a dropout rate of 10%, indicated that at least 22 subjects would be needed for each group. The sample size of each treatment group was estimated based on the differences and variations observed in a previous study by Evans et al. (2003).

Continuous variables with normal distribution (Height, Recovery time, HR, SBP, DBP, BIS) were presented as mean ± standard deviation, and continuous variables with non-normal distribution (Age, Weight, BMI, RDI, AHI, Onset time, DISE time, Operation duration, Residence time, RR and SpO_2_) were represented by median (interquartile range). Frequency (%) was used for categorical variables (Gender, UMSS score, ABJ score, severity of OSAHS, ASA physical status, Mallampati score, Percentage of success and AEs).

Continuous variables with normal distribution were compared between the two groups by independent sample *t* test, and these variables at different time points within each group were compared by repeated measure ANOVA. Continuous variables with non-normal distribution were compared between the two groups by Wilcoxon rank sum test. Non-rank categorical variables (Percentage of success, AEs) were tested by Chi-square test, and rank categorical variables (UMSS score, ABJ score, severity of OSAHS, ASA physical status, Mallampati score) were tested by Wilcoxon rank sum test. All statistical analysis was conducted with SPSS26 software. *P* < 0.05 was considered statistically significant.

## Results

### Demographic and polysomnography data

**FIGURE 1:**
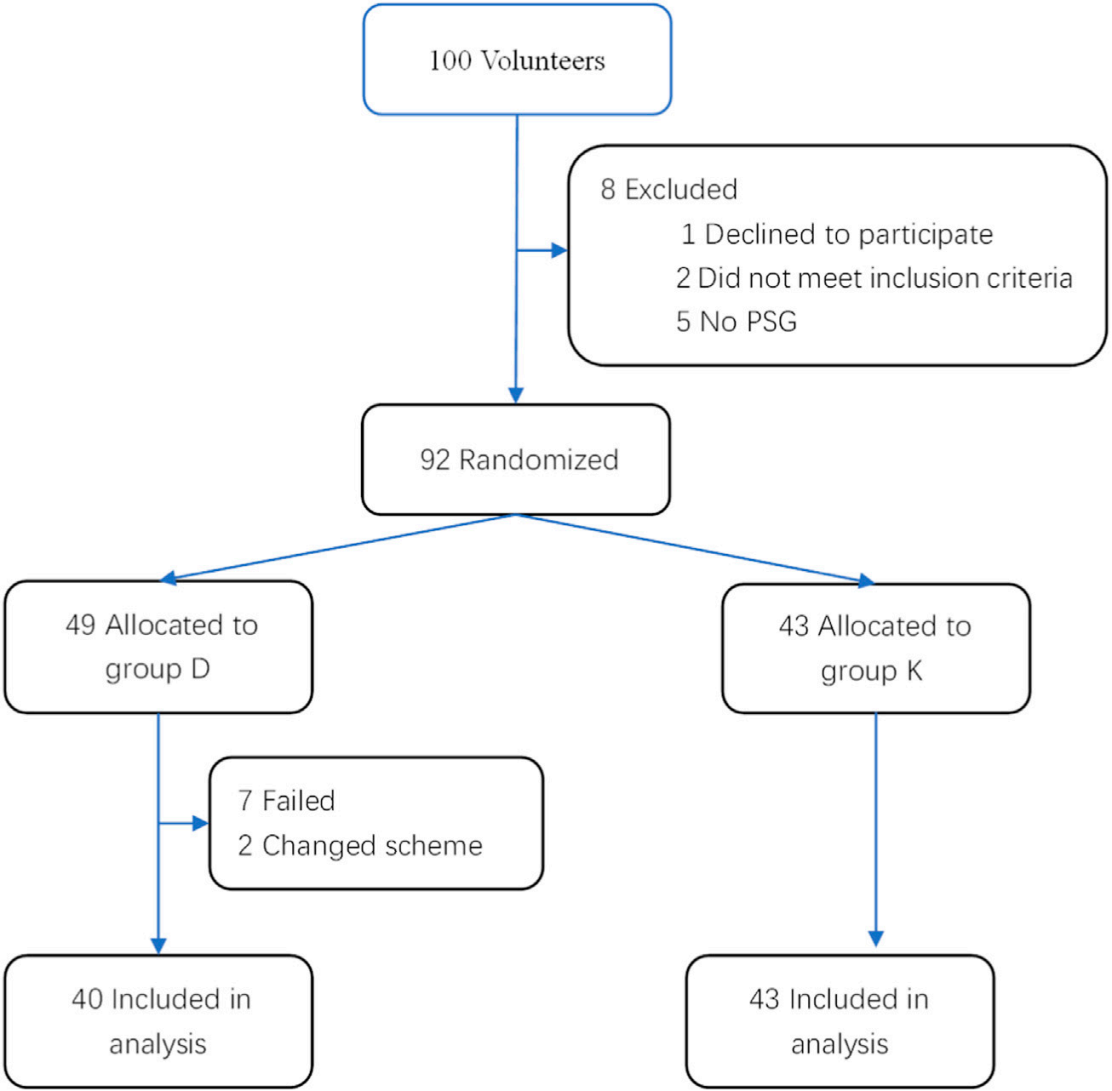
Flow diagram illustrating patients’ enrollment throughout the study.

100 children were enrolled in the study. Of them, 2 did not meet the inclusion criteria, 1 declined to participate, 5 had no PSG, 2 changed scheme, and 7 failed to undergo the complete DISE, and thus we dropped these 17 children from the study. Their data were not used in analysis, but the 7 children who had early termination of DISE were included in the calculation of percentage of success (Figure 1).

Demographic and PSG data had no difference between the two groups (*p* > 0.05). PSG monitoring demonstrated some degree of OSAHS on 68 subjects, of whom 57 had mild OSAHS, 6 had moderate OSAHS, and 5 had severe OSAHS (Table 1).

**TABLE 1:** Demographic and PSG data.

Characteristic	Total (*n* = 83)	Group D (*n* = 40)	Group K (*n* = 43)	*p*-value
Gender (n%)				0.600
Male	42 (50.6%)	19 (47.5%)	23 (53.5%)	
Female	41 (49.4%)	21 (52.5%)	20 (46.5%)	
Age(m)	79 (60.50–99)	70.50 (56.25–99)	83.50 (69.75–100.25)	0.433
Height (cm)	122.90 ± 15.37	119.94 ± 16.39	125.71 ± 13.94	0.100
Weight (kg)	24 (18–30)	21.00 (17.13–26.38)	25.50 (18.00–32.63)	0.050
BMI (kg.m-2)	15.53 (14.50–18.01)	15.38 (14.44–16.89)	16.08 (14.67–18.68)	0.183
RDI	1.4 (1.00–3.45)	1.25 (1.00–3.03)	1.65 (0.95–3.65)	0.600
AHI	1.4 (0.98–3.38)	1.25 (1.00–3.03)	1.65 (0.95–3.65)	0.498
Severity of OSAHS				0.426
No	15 (18.1%)	9 (22.5%)	6 (14.0%)	
I	57 (68.7%)	24 (60.0%)	33 (76.7%)	
II	6 (7.2%)	4 (10.0%)	2 (4.7%)	
III	5 (6.0%)	3 (7.5%)	2 (4.7%)	
ASA physical status				0.916
I	17 (20.5%)	8 (20.0%)	9 (20.9%)	
II	66 (79.52%)	32 (80.0%)	34 (79.1%)	
Mallampati Score				0.905
I	64 (77.11%)	30 (75.0%)	34 (79.1%)	
II	17 (20.48%)	9 (22.5%)	8 (18.6%)	
III	2 (2.41%)	1 (2.5%)	1 (2.33%)	

PSG, polysomnography; BMI, body mass index; RDI, respiratory disturbance index; AHI, Apnea-hypopnea Index. OSA, obstructive sleep apnea; ASA, American Society of Anesthesiologist. Data presented as mean ± SD, median (interquartile range) or frequency (%). p presented the comparison between the two groups. *p < 0.05, **p < 0.01, ***p < 0.001.

### Vital signs

**FIGURE 2:**
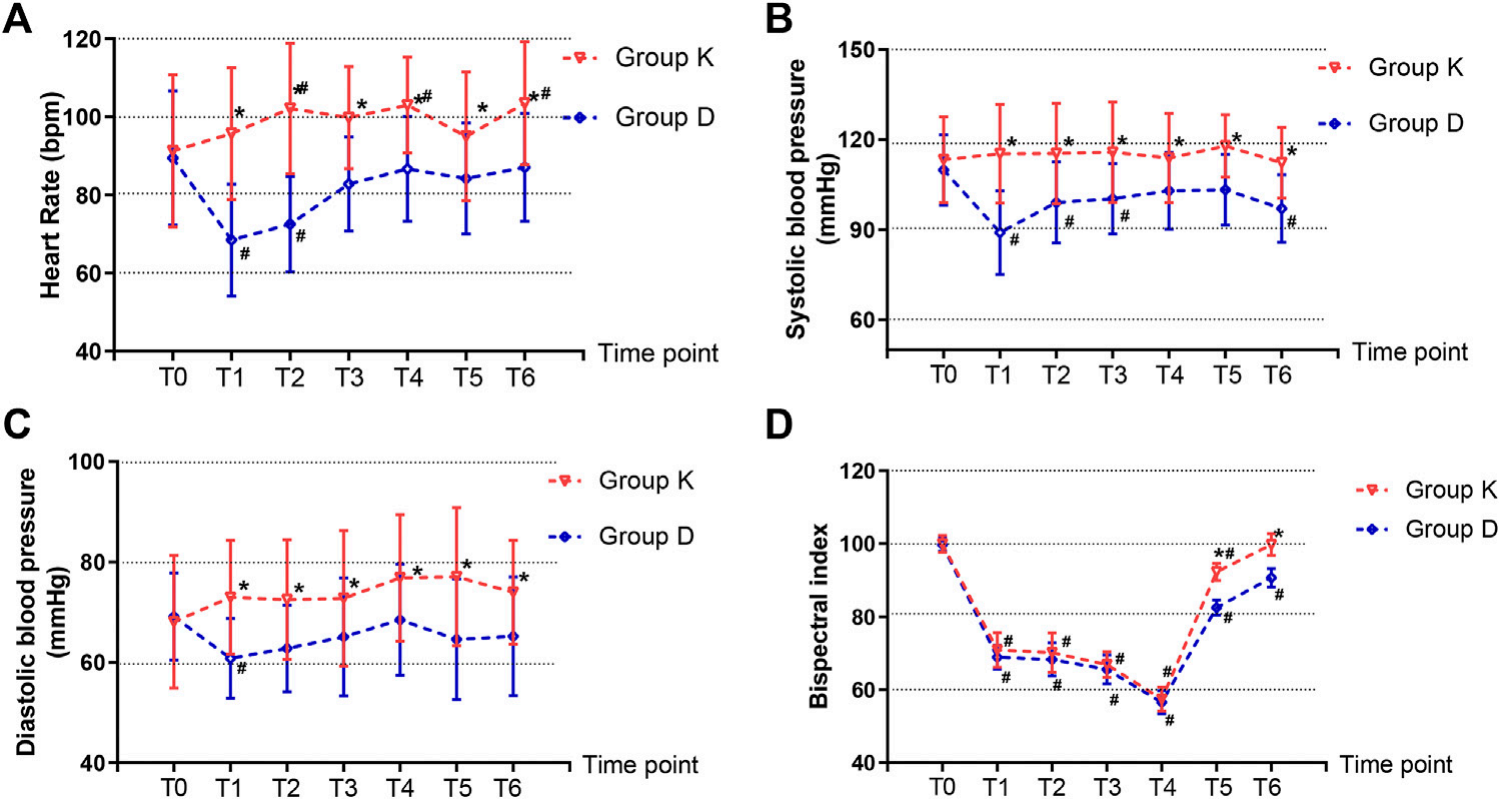
Hemodynamic changes and the bispectral index (BIS) records. **(A)** Heart rate (HR). **(B)** Systolic blood pressure (SBP). **(C)** Diastolic blood pressure (DBP). **(D)** The bispectral index (BIS). Data are expressed as mean ± SD, Compare between two groups *p < 0.05. Compare within time points of the same group ^#^ *p* < 0.05.

Baseline HR, SBP and DBP did not differ between the two groups. Compared with T0, SBP and DBP at T1 T2 T3 T4 T5 T6, and HR at T1 T3 T5 in Group K were not significantly different. Compared with T0, HR, SBP and DBP all decreased at T1 in Group D. Compared with Group D, children receiving esketamine had higher HR, SBP and DBP at T1,T2,T3,T4, T5, and T6 (Figures 2A,B,C,).

The SpO_2_ values recorded were 100% (95% CI, 99%–100%) for the esketamine group and 100% (95% CI, 98%–100%) for the dexmedetomidine group respectively. SpO_2_ values did not differ between the two groups (*p* = 0.135).

No abnormal ECG and RR were observed in both groups.

### Time for each procedure

Onset time, from starting of dexmedetomidine or esketamine infusion to DISE beginning, was 12 (95% CI, 10.25–14) min in Group D, and onset time of Group K was 2 (95% CI, 2–3) min. The onset time of Group K was shorter than that of Group D (*p* = 0.000) (Table 2) (Figure 4B).

**TABLE 2:** Time for each procedure.

Time (min)	Total	Group D	Group K	p-value
Onset (min)	3 (2–12)	12 (10.25–14)	2 (2–3)	0.000*
DISE(min)	12 (12–12)	12 (12–12)	12 (12–12)	0.851
Operation (min)	50 (35–60)	50 (40–60)	45 (35–60)	0.204
Recovery (min)	47.55 ± 16.639	54.18 ± 18.812	50.40 ± 17.389	0.344
Residence (min)	30 (30–37)	30 (30–35)	30 (30–40)	0.649

DISE, drug-induced sleep endoscopy. Data presented as mean ± SD, or median (interquartile range). Compare between two groups *p < 0.05, **p < 0.01, ***p < 0.001.

DISE time, from the beginning to the end of endoscopy, was 12.0 (95% CI, 12.0–12.0) min in both groups. DISE time had no significant difference between the two groups (Table 2) (*p* = 0.851).

The mean operation duration of the two groups, from the beginning to the end of the operation, was 50 (95% CI, 35–60) min. The operation duration had no significant difference between the two groups (Table 2) (*p =* 0.204).

Recovery time, from discontinuing of dexmedetomidine or esketamine infusion to eye opening on verbal contact, was 54.18 ± 18.81 min in Group D, and recovery time of Group K was 50.40 ± 17.39 min. The recovery time had no significant difference between the two groups (Table 2) (*p* = 0.344).

The mean residence time at PACU, from entering PACU to leaving PACU, was 30 (95%CI, 30–37) min. The residence-time at PACU had no significant difference between the two groups (Table 2) (*p* = 0.649).

### University of michigan sedation scale score, bispectral index and awakening and behavior judgment score

**FIGURE 3:**
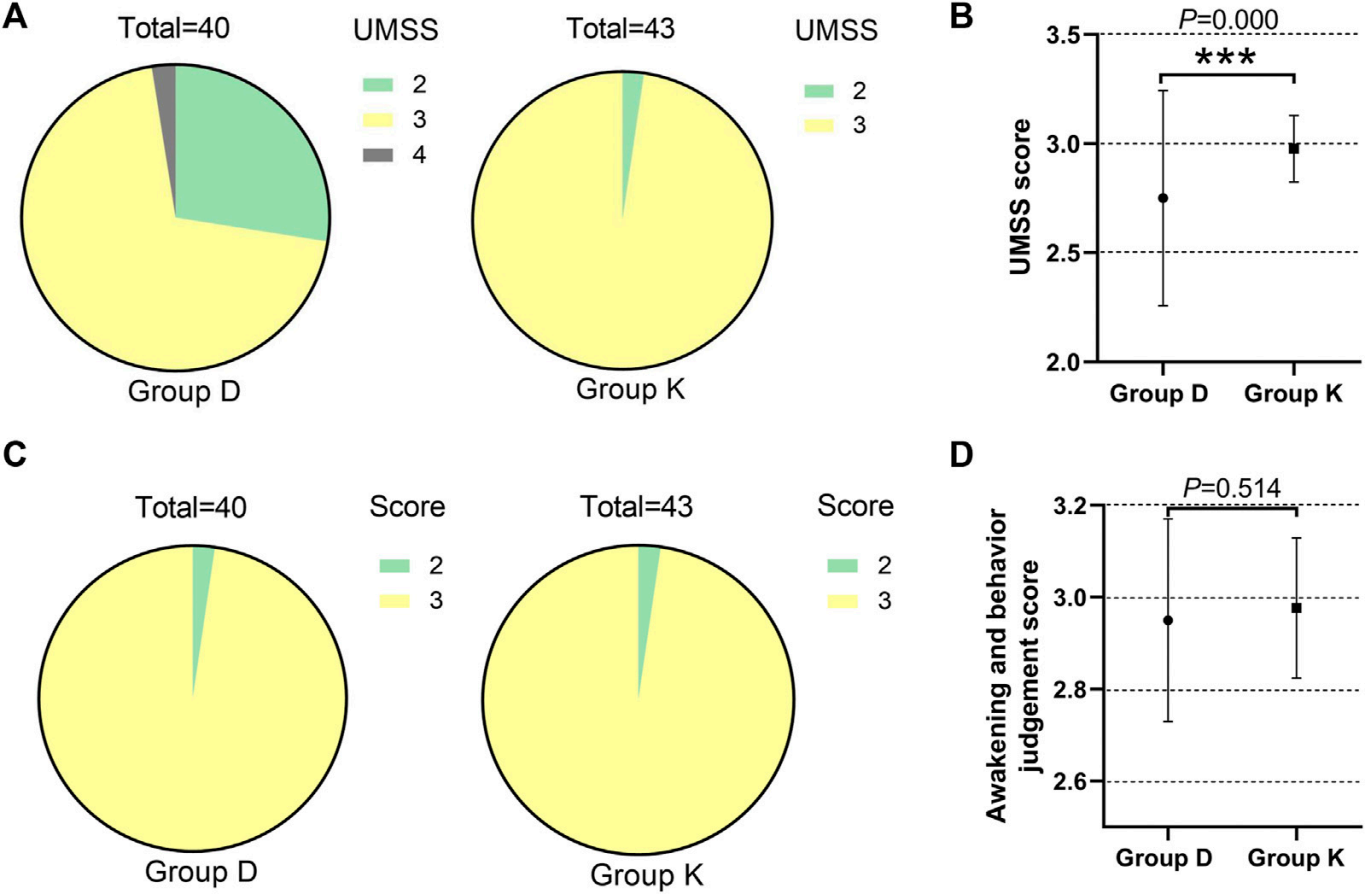
Different effects of two groups during drug-induced sleep endoscopy in children with obstructive sleep apnea hypopnea syndrome. **(A)**The number of subjects with each UMSS score in two groups. **(B)** UMSS score. **(C)**The number of subjects with each ABJ score in two groups. **(D)**ABJ score. Data are expressed as median (interquartile range) or frequency (%), Compare between two groups *p < 0.05, **p < 0.01, ***p < 0.001.

UMSS score: Depth of sedation was evaluated by UMSS after completion of bolus dose administration. Most subjects had a UMSS score of 2 or 3 at the time of evaluation. The number of subjects with each score varied between the two groups. For the subjects receiving dexmedetomidine, 11 had a score of 2 and 28 had a score of 3. For the subjects receiving esketamine, 1 had a score of 2, and 42 had a score of 3. Group K had a higher UMSS score than Group D (*p* = 0.005) (Table 3) (Figures 3A,B).

**TABLE 3:** UMSS score and Awakening and behavior judgment (ABJ) score.

Score	Total (83)	Group D (40)	Group K (43)	p-value
Awakening and behavior judgment				0.514
2	3 (3.61%)	2 (5.00%)	1 (2.33%)	
3	80 (96.39%)	38 (95.00%)	42 (97.6%)	
UMSS				0.005**
2	12 (14.46)	11 (27.50%)	1 (2.33%)	
3	70 (84.34)	28 (70.00%)	42 (97.67%)	
4	1 (1.20%)	1 (2.50%)	0 (0.00%)	

UMSS: University of Michigan Sedation Scale. Data presented as frequency (%). Group D vs. Group K *p < 0.05, **p < 0.01, ***p < 0.001.

BIS: BIS at T0, T1, T2, T3 and T4 had no significant difference between the two groups. Group K had higher BIS at T5 and T6 than Group D (Figure 2D).

ABJ score: All subjects had an ABJ score of 2 or 3. The number of subjects with each score varied between the two groups. Of the subjects receiving dexmedetomidine, 2 had a score of 2 and 38 had a score of 3. And of the subjects receiving esketamine, 1 had a score of 2 and 42 had a score of 3. The ABJ scores didn’t vary between the two groups (*p* = 0.514) (Table 3) (Figures 3C,D).

### Adverse effects, propofol rescue and percentage of success

**FIGURE 4:**
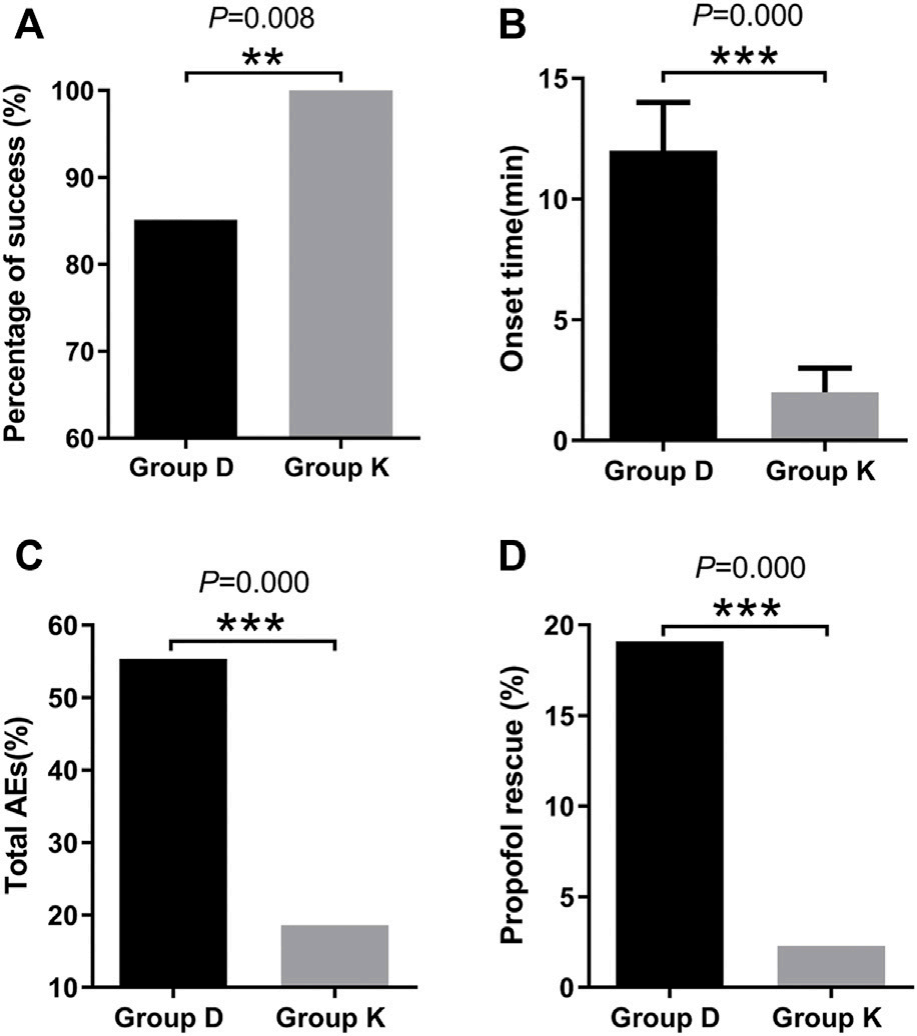
Different effects of two groups during drug-induced sleep endoscopy in children with obstructive sleep apnea hypopnea syndrome. **(A)**Percentage of success. **(B)**Onset time. **(C)**Total AEs. **(D)**Propofol rescue. Data are expressed as median (interquartile range) or frequency (%), Compare between two groups *p < 0.05, **p < 0.01, ***p < 0.001.

AEs: In Group D, 26 of 47 patients experienced AEs, and 8 of 43 patients experienced AEs in Group K. The number of AEs varied greatly between the two groups (*p* = 0.000). The risk of AEs and propofol rescue was higher in Group D than in Group K (*p* = 0.000) (Table 4) (Figures 4C,D).

**TABLE 4:** Adverse effects, Propofol rescue and Percentage of success.

Symptoms	D group (*n* = 47)	K group (*n* = 43)	*p*-value
Patient movement	14 (29.8%)	0 (0.0%)	
Cry	2 (4.3%)	1 (2.3%)	
Sleepy	3 (6.4%)	0 (0.0%)	
Laryngospasm	1 (2.1%)	0 (0.0%)	
Hypoxemia	4 (8.5%)	6 (14.0%)	
Allergy	1 (2.1%)	0 (0.0%)	
PONV	0 (0.0%)	0 (0.0%)	
Sore throat	0 (0.0%)	0 (0.0%)	
Nystagmus	0 (0.0%)	0 (0.0%)	
Hypertension	0 (0.0%)	0 (0.0%)	
Delirium	0 (0.0%)	0 (0.0%)	
Coughing	1 (2.1%)	0 (0.0%)	
Salivation	0 (0.0%)	1 (2.3%)	
Overnight respiratory events	0 (0.0%)	0 (0.0%)	
Related to drugs			
Certainly	23 (48.9%)	6 (14.0%)	
Probably	3 (6.4%)	2 (4.7%)	
Total	26 (55.3%)	8 (18.6%)	0.000***
propofol rescue	9 (19.1%)	1 (2.3%)	0.000***
Percentage of success	40 (85.11%)	43 (100%)	0.008**

PONV, postoperative nausea and vomiting. Data presented as frequency (%). Group D vs. Group K *p < 0.05, **p < 0.01, ***p < 0.001.

Percentage of success: The ratios of completed DISE cases and total cases, were 85.11% (40/47) in Group D and 100% (43/43) in Group K respectively. Overall, DISE was successfully completed in 92.22% (83/90) of cases. In Group D, DISE was not completed in 7 patients. Group K had a higher percentage of success than Group D (*p* = 0.008) (Table 4) (Figure 4A).

## Discussion

In this prospective, randomized and blinded clinical trial, esketamine iv can provide higher percentage of success, shorter onset time, deeper sedation and fewer AEs, therefore it is superior to dexmedetomidine iv.

It can be seen from this study that the respective percentage of success was 85.11% (40/47) in Group D and 100% (43/43) in Group K. Since 7 patients failed to undergo the complete DISE in Group D, Group K had a higher percentage of success than Group D (*p* = 0.008). The reasons for increased risk of DISE failure of Group D were probably delayed onset and slighter sedation.

Our findings revealed that esketamine had quicker onset than dexmedetomidine. The respective onset time was 12 (95% CI, 10.25–14) min in Group D and 2 (95% CI, 2–3) min in Group K, which was consistent with Pees’ (Pees et al., 2003) and Tekeli’s research (Tekeli et al., 2020). Our difference from Pee’s research (Pees et al., 2003) was the subjects were adults in Pee’s but children in ours. Our study showed no significant difference in recovery time between the two groups (*p* = 0.344). It was probably because drugs including dexmedetomidine and esketamine had been eliminated during the whole period of DISE followed by adenotonsillectomy.

The UMSS score of Group K was higher than that of Group D, which proved that esketamine had preferred sedation. This result was similar to Pees’ (Pees et al., 2003). Contrary to the previous studies made by Lo YL and Haberland CM (Haberland et al., 2011; Lo et al., 2015), difference in BIS between the two groups at T0, T1, T2, T3, and T4 were not significant. The BIS was one of the most accurate and sensitive indicators of accessing conscious state by a single numeric value, scaled from 0 to 100 (Lo et al., 2015; Jones et al., 2021). However, according to Ibrahim AE (Ibrahim et al., 2001), BIS scores associated with depth of anesthesia were dependent upon the anesthetic agent being used. They were relatively ineffective during sedation with ketamine, nitrous oxide, or dexmedetomidine, and could be unpredictable in the presence of opioids. Further research was needed.

AEs occurred in 8 of 43 patients in Group K and 26 of 47 patients in Group D. The risk of AEs was proved to be lower in Group K (*p* = 0.000).

No abnormal ECG and RR were observed in both groups. SpO_2_ between the two groups had no significant difference (*p* = 0.135). It was shown that neither dexmedetomidine nor esketamine had airway intervention or significant oxygen desaturation. This gave further support to earlier experimental results (Mahmoud et al., 2009; Ehsan et al., 2016). Compared with T0, HR, SBP and DBP all decreased at T1 in Group D. In accordance with Nelson’s research (Nelson et al., 2003), dexmedetomidine might cause marked hemodynamic instability, especially bradycardia and hypotension.

Compared with T0, SBP and DBP at T1 T2 T3 T4 T5 T6, and HR at T1 T3 T5 in Group K were not significantly different. Our research revealed for the first time that esketamine, administered along with midazolam, had little influence on the circulatory and respiratory system during OSAHS pediatric DISE.

The pharmacokinetic parameters of esketamine and S-norketamine are both similar in the pure isomer and the racemate. Also, there were no sex differences in the pharmacokinetics of esketamine and S-norketamine in the pure isomer. However, compared with racemate ketamine, esketamine had a shorter recovery time and orientation recovery time, which present potential clinical advantages (Wang et al., 2019). Esketamine possesses a higher efficiency and mainly acts on N-methyl-D-aspartate (NMDA) receptor and integrates sedation, analgesia, and the anesthesia effect (Smits et al., 2017; Van de Bunt et al., 2017; Kalmoe et al., 2020; Harder et al., 2022; Li et al., 2022). Its analgesic effect is twice that of ketamine; therefore, lower clinical doses of esketamine are demanded, and side effects (such as nightmare, delirium, and agitation) are decreased (Zhang et al., 2022). It has been adopted in some European countries for decades and has been used in Chinese hospitals in recent years. Besides treating depression, esketamine is applied for clinical sedation and analgesia associated with same-day bidirectional endoscopy (Long et al., 2022), pediatric dental surgery (Xin et al., 2021) and mechanical ventilation in ICU patients (Song et al., 2022) When it is used for bronchoscopy, esketamine relaxes bronchiolar muscles and inhibits bronchial constriction (Huang et al., 2022).

The following are several limitations of our research:

First, the DISE technique doesn’t reliably induce REM sleep, which is closely connected with upper airway obstruction (Capasso et al., 2016);

Second, the depth of sleep and wakefulness cannot be assessed effectively in an accurate and consistent way. Based on previous research, adequate depth of sedation and anesthesia for DISE in OSAHS children was UMSS score of 3 or BIS 65–75 in this study (Stierer and Ishman, 2015; Lo et al., 2015; Shields et al., 2005; Malviya et al., 2002). The scores for pediatric awakening depth were very few. A reliable and valid one was ABJ score for newborns and children (Pees et al., 2003). So UMSS score, BIS and ABJ score were adopted in our study.

Third, this study, carried out at a clinical DISE center specializing in the care of OSAHS children, is only a reflection of the experience of a single center.

Fourth, in the absence of dose-response studies, we cannot determine the effect of using larger or smaller doses in our study.

Fifth, in our study DISE was not done alone but performed before adenotonsillectomy.

Last but not least, the majority of pediatric patients in our study have mild OSAHS or even suspected ones.

Further research on dosage related effects of esketamine for pediatric DISE needs to be done at multiple centers. The relationship between BIS scores and depth of anesthesia under esketamine for OSAHS pediatric DISE should be explored.

## Conclusion

Esketamine in comparison to dexmedetomidine provides more effective and safer depth of anesthesia for OSAHS pediatric DISE by ensuring short onset time, deep sedation, and few AEs.

## Data Availability

The original contributions presented in the study are included in the article/supplementary material, further inquiries can be directed to the corresponding authors.

## Abbreviations

OSAHS, obstructive sleep apnea hypopnea syndrome; PSG, polysomnography; BMI, Body Mass Index; RDI, respiratory disturbance index; AHI, Apnea-hypopnea Index; ASA, American Society of Anesthesiologists; DISE, drug-induced sleep endoscopy; UMSS, University of Michigan Sedation Scale; ABJ score, Awakening and behavior judgment score for newborns and children; PONV, postoperative nausea and vomiting; BIS, bispectral index; ECG, electrocardiogram; HR, heart rate; SBP, systolic blood pressure; DBP, diastolic blood pressure; RR, respiratory rate; SpO2, pulse oxygen saturation; AEs, adverse effects; SD, standard deviation; ANOVA, one-way analysis of variance.

## References

[B1] Bhattacharjee R. Kheirandish-Gozal L. Spruyt K. Mitchell R. B. Promchiarak J. N., (2010). Adenotonsillectomy outcomes in treatment of obstructive sleep apnea in children: A multicenter retrospective study. Am. J. Respir. Crit. Care Med. 182 (5), 676–683. 10.1164/rccm.200912-1930OC 20448096

[B2] Capasso R. Rosa T. Tsou D. Y. Nekhendzy D. Drover J. Collins S. (2016). Variable findings for drug-induced sleep endoscopy in obstructive sleep apnea with propofol versus dexmedetomidine. Otolaryngol. Head. Neck Surg. 154 (4), 765–770. 10.1177/0194599815625972 26814208

[B3] Cho J. S. Soh S. Kim E. J. Cho H. J. Shin S. Kim H. J. (2015). Comparison of three sedation regimens for drug-induced sleep endoscopy. Sleep. Breath. 19 (2), 711–717. 10.1007/s11325-015-1127-9 25643766

[B4] Ehsan Z. Mahmoud M. Shott S. R. Amin R. S. Ishman S. L. (2016). The effects of anesthesia and opioids on the upper airway: A systematic review. Laryngoscope 126 (1), 270–284. 10.1002/lary.25399 26198715

[B5] Evans R. G. Crawford M. W. Noseworthy M. D. Yoo S. J. (2003). Effect of increasing depth of propofol anesthesia on upper airway configuration in children. Anesthesiology 99 (3), 596–602. 10.1097/00000542-200309000-00014 12960543

[B6] Goldbart A. D. Levitas A. Greenberg-Dotan S. Ben Shimol A. Broides M. Puterman M. (2010). B-type natriuretic peptide and cardiovascular function in young children with obstructive sleep apnea. Chest 138 (3), 528–535. 10.1378/chest.10-0150 20558551

[B7] Haberland C. M. Baker S. Liu H. (2011). Bispectral index monitoring of sedation depth in pediatric dental patients. Anesth. Prog. 58 (2), 66–72. 10.2344/0003-3006-58.2.66 21679042 PMC3198129

[B8] Harder M. Fiegl-Lechner A. Oberacher H. Horvath U. E. I. Schlager A. Jeske M. (2022). Stability evaluation of morphine, hydromorphone, metamizole and esketamine containing analgesic mixtures applied for patient-controlled analgesia in hospice and palliative care. Biomed. Chromatogr. 36 (4), e5340. 10.1002/bmc.5340 35043434 PMC9285503

[B9] Huang X. Ai P. Wei C. Sun Y. Wu A. (2022). Comparison of the effects of esketamine/propofol and sufentanil/propofol on the incidence of intraoperative hypoxemia during bronchoscopy: Protocol for a randomized, prospective, parallel-group trial. J. Clin. Med. 11 (15), 4587. 10.3390/jcm11154587 35956202 PMC9369459

[B10] Ibrahim A. E. Taraday J. K. Kharasch E. D. (2001). Bispectral index monitoring during sedation with sevoflurane, midazolam, and propofol. Anesthesiology 95 (5), 1151–1159. 10.1097/00000542-200111000-00019 11684984

[B11] Jones J. H. Nittur V. R. Fleming N. Applegate R. L. (2021). Simultaneous comparison of depth of sedation performance between SedLine and BIS during general anesthesia using custom passive interface hardware: Study protocol for a prospective, non-blinded, non-randomized trial. BMC Anesthesiol. 21 (1), 105. 10.1186/s12871-021-01326-5 33823811 PMC8022390

[B12] Kalmoe M. C. Janski A. M. Zorumski C. F. Nagele P. Palanca B. J. Conway C. R. (2020). Ketamine and nitrous oxide: The evolution of NMDA receptor antagonists as antidepressant agents. J. Neurol. Sci. 412, 116778. 10.1016/j.jns.2020.116778 32240970

[B13] Kelly A. Dougherty S. Cucchiara A. Marcus C. L. Brooks L. J. (2010). Catecholamines, adiponectin, and insulin resistance as measured by HOMA in children with obstructive sleep apnea. Sleep 33 (9), 1185–1191. 10.1093/sleep/33.9.1185 20857865 PMC2938859

[B14] Li A. M. So H. K. Au C. T. Ho C. Lau J. Ng S. K. (2010). Epidemiology of obstructive sleep apnoea syndrome in Chinese children: A two-phase community study. Thorax 65 (11), 991–997. 10.1136/thx.2010.134858 20965935

[B15] Li X. Xiang P. Liang J. Deng Y. Du J. (2022). Global trends and hotspots in esketamine research: A bibliom etric analysis of past and estimation of future trends. Drug Des. devel. Ther. 16, 1131–1142. 10.2147/DDDT.S356284 PMC9037742 35478936

[B16] Liu K. A. Liu C. C. Alex G. Szmuk P. Mitchell R, B. (2020). Anesthetic management of children undergoing drug-induced sleep endoscopy: A retrospective review. Int. J. Pediatr. Otorhinolaryngol. 139 (12), 110440. 10.1016/j.ijporl.2020.110440 33080472

[B17] Lo Y. L. Ni Y. L. Wang T. Y. Lin H. Y. Li D. P. White J. R. (2015). Bispectral index in evaluating effects of sedation depth on drug-induced sleep endoscopy. J. Clin. Sleep. Med. 11 (9), 1011–1020. 10.5664/jcsm.5016 25979098 PMC4543245

[B18] Long Y. Q. Feng C. D. Ding Y. Y. Feng X. M. Liu H. Ji F. H. (2022). Esketamine as an adjuvant to ciprofol or propofol sedation for same-day bidirectional endoscopy: Protocol for a randomized, double-blind, controlled trial with factorial design. Front. Pharmacol. 3 (13), 821691. 10.3389/fphar.2022.821691 PMC8975265 35370640

[B19] Mahmoud M. Gunter J. Donnelly L. F. Wang Y. Nick T. G. Sadhasivam S. (2009). A comparison of dexmedetomidine with propofol for magnetic resonance imaging sleep studies in children. Anesth. Analg. 109 (3), 745–753. 10.1213/ane.0b013e3181adc506 19690241

[B21] Mahmoud M. Mason K. P. (2015). Dexmedetomidine: Review, update, and future considerations of paediatric perioperative and periprocedural applications and limitations. Br. J. Anaesth. 115 (2), 171–182. 10.1093/bja/aev226 26170346

[B22] Malviya S. Voepel-Lewis T. Tait A. R. Merkel S. Tremper K. Naughton N. (2002). Depth of sedation in children undergoing computed tomography: Validity and reliability of the university of Michigan sedation Scale (UMSS). Br. J. Anaesth. 88 (2), 241–245. 10.1093/bja/88.2.241 11878656

[B23] Marcus C. L. Brooks L. J. Draper K. A. Gozal D. Halbower A. C. Jones J. (2012). Diagnosis and management of childhood obstructive sleep apnea syndrome. Pediatrics 130 (3), e714–e755. 10.1542/peds.2012-1672 22926176

[B24] Martin C. S. Deverman S. E. Norvell D. C. Cusick J. C. Kendrick A. Koh J. (2019). Randomized trial of acupuncture with antiemetics for reducing postoperative nausea in children. Acta Anaesthesiol. Scand. 63 (3), 292–297. 10.1111/aas.13288 30397904

[B25] Miano S. Paolino M. C. Urbano A. Parisi P. Massolo A. C. Castaldo R. (2011). Neurocognitive assessment and sleep analysis in children with sleep-disordered breathing. Clin. Neurophysiol. 122 (2), 311–319. 10.1016/j.clinph.2010.06.019 20637692

[B26] Murphy M. Bruno M. A. Riedner B. A. Boveroux P. Noirhomme Q. Landsness E. C. (2011). Propofol anesthesia and sleep: a high-density EEG study. Sleep 34 (3), 283–91A. 10.1093/sleep/34.3.283 21358845 PMC3041704

[B27] Nelson L. E. Lu J. Guo T. Saper C. B. Franks N. P. Maze M. (2003). The alpha2-adrenoceptor agonist dexmedetomidine converges on an endogenous sleep-promoting pathway to exert its sedative effects. Anesthesiology 98 (2), 428–436. 10.1097/00000542-200302000-00024 12552203

[B28] Park J. G. Ramar K. Olson E, J. (2011). Updates on definition, consequences, and management of obstructive sleep apnea. Mayo Clin. Proc. 86 (6), 549–554. quiz 554-555. 10.4065/mcp.2010.0810 21628617 PMC3104914

[B29] Pees C. Haas N. A. Ewert P. Berger F. Lange P. E. (2003). Comparison of analgesic/sedative effect of racemic ketamine and S(+)-ketamine during cardiac catheterization in newborns and children. Pediatr. Cardiol. 24 (5), 424–429. 10.1007/s00246-002-0356-4 14627307

[B30] Schmidt A. Oye I. Akeson J. (2005). Cerebral physiological responses to bolus injection of racemic, S(+)- or R(-)-ketamine in the pig. Acta Anaesthesiol. Scand. 49 (10), 1436–1442. 10.1111/j.1399-6576.2005.00838.x 16223386

[B31] Shields C. H. Styadi-Park G. McCown M. Y. Creamer K. M. (2005). Clinical utility of the bispectral index score when compared to the University of Michigan Sedation Scale in assessing the depth of outpatient pediatric sedation. Clin. Pediatr. 44 (3), 229–236. 10.1177/000992280504400306 15821847

[B32] Smith D. F. He S. Peddireddy N, S. Vairavan Manickam P. C. Heubi H. Shott S. R. (2020). Effectiveness of pediatric drug-induced sleep endoscopy for REM-predominant obstructive sleep apnea. Sleep. Breath. 24 (4), 1705–1713. 10.1007/s11325-020-02056-7 32277395 PMC8007082

[B33] Smits G. J. Kuypers M. I. Mignot L. A. Reijners E. P. Oskam E. Doom K. V. (2017). Procedural sedation in the emergency department by Dutch emergency physicians: A prospective multicentre observational study of 1711 adults. Emerg. Med. J. 34 (4), 237–242. 10.1136/emermed-2016-205767 27797871 PMC5502244

[B34] Song X. Wang F. Dong R. Zhu K. Wang C. (2022). Efficacy and safety of remimazolam tosilate combined with esketamine for analgesic sedation in mechanically ventilated ICU patients: A single-arm clinical study protocol. Front. Med. 9, 832105. 10.3389/fmed.2022.832105 PMC8968316 35372406

[B35] Sruthi S. Mandal B. Rohit M. K. Puri G. D. (2018). Dexmedetomidine versus ketofol sedation for outpatient diagnostic transesophageal echocardiography: A randomized controlled study. Ann. Card. Anaesth. 21 (2), 143–150. 10.4103/aca.ACA_171_17 29652275 PMC5914214

[B36] Stierer T. L. Ishman S. L. (2015). Bispectral index in evaluating effects of sedation depth on drug-induced sleep endoscopy: DISE or No dice. J. Clin. Sleep. Med. 11 (9), 965–966. 10.5664/jcsm.5002 26285116 PMC4543255

[B37] Tekeli A. E. Oguz A. K. Tuncdemir Y. E. Almali N. (2020). Comparison of dexmedetomidine-propofol and ketamine-propofol administration during sedation-guided upper gastrointestinal system endoscopy. Med. Baltim. 99 (49), e23317. 10.1097/MD.0000000000023317 PMC7717792 33285707

[B38] van de Bunt J. A. Veldhoen E. S. Nievelstein R. A. J. Hulsker C. C. C. Schouten A. N. J. van Herwaarden M. Y. A. (2017). Effects of esketamine sedation compared to morphine analgesia on hydrostatic reduction of intussusception: A case-cohort comparison study. Paediatr. Anaesth. 27 (11), 1091–1097. 10.1111/pan.13226 28940868

[B39] Wang J. Huang J. Yang S. Cui C. Ye L. Wang S. Y. (2019). Pharmacokinetics and safety of esketamine in Chinese patients undergoing painless gastroscopy in comparison with ketamine: A randomized, open-label clinical study. Drug Des. devel. Ther. 13, 4135–4144. 10.2147/DDDT.S224553 PMC6902860 31827320

[B40] Xin N. Xu H. Yue C. (2021). Comparison between dexmedetomidine and esketamine in pediatric dentistry surgery. Transl. Pediatr. 10 (12), 3159–3165. 10.21037/tp-21-435 35070829 PMC8753461

[B41] Zhang C. He J. Shi Q. Bao F. Xu J. (2022). Subanaesthetic dose of esketamine during induction delays anaesthesia recovery a randomized, double-blind clinical trial. BMC Anesthesiol. 22 (1), 138. 10.1186/s12871-022-01662-0 35534825 PMC9082902

